# Land Use, anthropogenic disturbance, and riverine features drive patterns of habitat selection by a wintering waterbird in a semi-arid environment

**DOI:** 10.1371/journal.pone.0206222

**Published:** 2018-11-07

**Authors:** Matthew A. Boggie, Daniel P. Collins, J. Patrick Donnelly, Scott A. Carleton

**Affiliations:** 1 Department of Biology, New Mexico State University, Las Cruces, New Mexico, United States of America; 2 U.S. Fish and Wildlife Service, Division of Migratory Birds, Albuquerque, New Mexico, United States of America; 3 Intermountain West Joint Venture and U.S. Fish and Wildlife Service, University of Montana, Missoula, Montana, United States of America; Consejo Superior de Investigaciones Cientificas, SPAIN

## Abstract

River ecosystems in semi-arid environments provide an array of resources that concentrate biodiversity, but also attract human settlement and support economic development. In the southwestern United States, land-use change, drought, and anthropogenic disturbance are compounding factors which have led to departures from historical conditions of river ecosystems, consequently affecting wildlife habitat, including important wintering areas for migratory birds. The Rio Grande (River) in central New Mexico is the lifeblood of the Middle Rio Grande Valley (MRGV), maintaining large urban and agricultural centers and riparian and wetland resources, which disproportionately support a diversity of wildlife. The MRGV has been identified as the most important wintering area for the Rocky Mountain Population of greater sandhill cranes (*Antigone canadensis tabida*). Presently, however, changes in the hydrogeomorphology of the Rio Grande and landscape modification by humans have reshaped the MRGV and winter habitat for sandhill cranes. To evaluate these impacts, we investigated how land-use practices, anthropogenic disturbance, and river morphology influenced patterns of diurnal and roosting habitat selection by sandhill cranes. During the diurnal period, sandhill cranes relied heavily on managed public lands selecting agriculture crops, such as corn fields, and wetlands for foraging and loafing while avoiding areas with increasing densities of human structures. Sandhill cranes selected areas for roosting in the Rio Grande characterized by shallower water interspersed with sandbars, wide channel width, low bank vegetation, and farther away from disturbances associated with bridges. Our results establish and identify the central processes driving patterns of diel habitat selection by wintering sandhill cranes. Land use and riverine trends have likely gradually reduced winter habitat to managed public lands and limited reaches of the Rio Grande, underscoring the importance of natural resources agencies in supporting migratory birds and challenges involved when managing for wildlife in highly pressured semi-arid environments.

## Introduction

Human-induced environmental change has increased the vulnerability of ecosystems globally and created uncertainty in their long-term sustainability and resilience [1–4]. Ecosystems in arid and semi-arid environments are particularly sensitive to variations in temperature, precipitation and natural resource overexploitation [5–8]. Accelerated land-use change in the arid southwestern United States coupled with drought, have compromised ecosystems services supporting a diversity of species [9–11]. Largely driven by human activities, landscape fragmentation, increasing water scarcity, soil erosion, and productivity loss have broad-scale impacts on humans and wildlife [12–14]. Moreover, in water limited environments, water may be sourced from a single drainage basin where urban, industrial, and agricultural demands place tremendous pressure on already over-burdened systems often jeopardizing resources wildlife require.

The Middle Rio Grande Valley (hereafter MRGV) is a semi-arid region in central New Mexico, USA transected by the Rio Grande (River). Since before European settlement, humans have gravitated to the MRGV to capitalize on water resources of the Rio Grande and fertile soils of its floodplain. Currently, the MRGV maintains large urban and agricultural centers and supports high biodiversity, including multiple threatened and endangered species. Unfortunately, the MRGV is a basin facing increased imperilment by way of human population growth compounded by impacts of climatic fluctuations. Long-term perturbations facilitated by changes in land use, high demands placed on limited water resources, introduction of invasive species, and human-modification of the natural hydrological regime has made it harder to simultaneously fulfill the needs of humans and wildlife [5, 15]. These environmental stressors have substantially changed both physiognomy and function of the MRGV and raised concern for the persistence of some fish and wildlife species [16–19].

Many wildlife species rely on wetland and riparian habitats along rivers in semi-arid environments and some are dependent on associated agricultural resources situated along river floodplains. Migratory birds, in particular, are reliant on the Rio Grande corridor to meet energetically demanding life-cycle events in an otherwise xeric and unproductive landscape [16, 20–23]. From October to February, the MRGV supports hundreds of thousands of overwintering waterbirds. Quality and availability of wintering habitat is an important driver of migratory bird populations and has been linked to timing of, and body condition during, spring migration which can subsequently effect reproductive success [24, 25]. Furthermore, many species of migratory birds have high fidelity to wintering grounds, consistently reusing the same general areas annually thus relying on permanency of habitat [26–28]. Consequently, any dramatic changes in habitat can have repercussions on population dynamics and possible cross-seasonal or carry-over effects that can influence demographics [29–31].

The MRGV is identified as the single most important overwinter area for the Rocky Mountain Population of greater sandhill cranes (*Antigone canadensis tabida*; hereafter sandhill cranes) [32, 33]. The geographic range of this population (~22,000 birds) spans the intermountain west, USA, with 80% of birds wintering in the MRGV [32, 34]. Despite population reliance on the MRGV, winter habitat requirements for sandhill cranes have yet to be quantitatively assessed [33]. Winter habitat for sandhill cranes can be characterized by riparian areas, and wetlands, such as moist-soil managed wetlands, proximal to irrigated agriculture and pastures [35]. The food resource base consists primarily of cultivated grains, such as corn, plant-based foods in wetlands, and a range of small vertebrates and macroinvertebrates [36, 37]. Shifts in agricultural patterns and landscape change, however, may be in conflict with the habitat needs of wintering sandhill cranes in the MRGV.

Drought, flood control, water diversion or damming, and human water consumption have drastically altered the natural hydrology of the Rio Grande [15], influencing habitat availability for sandhill cranes. Transition away from grain-based crops, an important dietary component for wintering sandhill cranes [38], have likely influenced the availability and quality of food resources [39, 40]. Several national wildlife refuges and state managed properties that have grown into important public lands serve a dual purpose within the MRGV; they support migratory birds throughout winter and mitigate human-wildlife conflicts by reducing crop depredation by sandhill cranes on private lands. The use of these properties by wintering sandhill cranes has been documented [33, 35, 37], but it is unclear how broader changes in water availability, urban encroachment, and private lands agriculture are influencing resource and sandhill crane distributions.

Establishing patterns of habitat selection for any species requires knowledge of distribution of, and variation in, resource availability to identify important habitat-related features and potential limiting factors. Measuring the effects that resource availability has on the behavior of sandhill cranes can lead to better understanding of how landscape change may influence population-level processes [41]. Furthermore, sandhill cranes can serve as an umbrella species, such that other species in the ecological community (e.g., waterfowl) benefit from associated conservation. By understanding how sandhill cranes respond to anthropogenic disturbance and natural processes that effect winter habitat, greater insight can be gained and leveraged to provide support for prioritizing wildlife management and conservation efforts in the MRGV.

It was our objective to examine space use patterns of sandhill cranes in the MRGV to determine how current landscape structure and composition are impacting winter habitat selection. Specifically we were interested in: 1) establishing current land use practices, land ownership, and level of human disturbance; 2) relating these to movement patterns of sandhill cranes to determine factors that influence selection of foraging and loafing habitat; 3) delineating river morphology and vertical vegetation structure of the riverbanks of the Rio Grande and establishing the connection of these to roost sites used by sandhill cranes; and 4) using predictive models to determine landscape features that define winter habitat for sandhill cranes and identify areas with high habitat value in the MRGV. Our results will provide a focused evaluation of how sandhill cranes are responding to current disturbances and a template for conditions on the wintering grounds that need to be maintained or enhanced to satisfy winter habitat requirements.

## Materials and methods

### Study area

**Fig 1:**
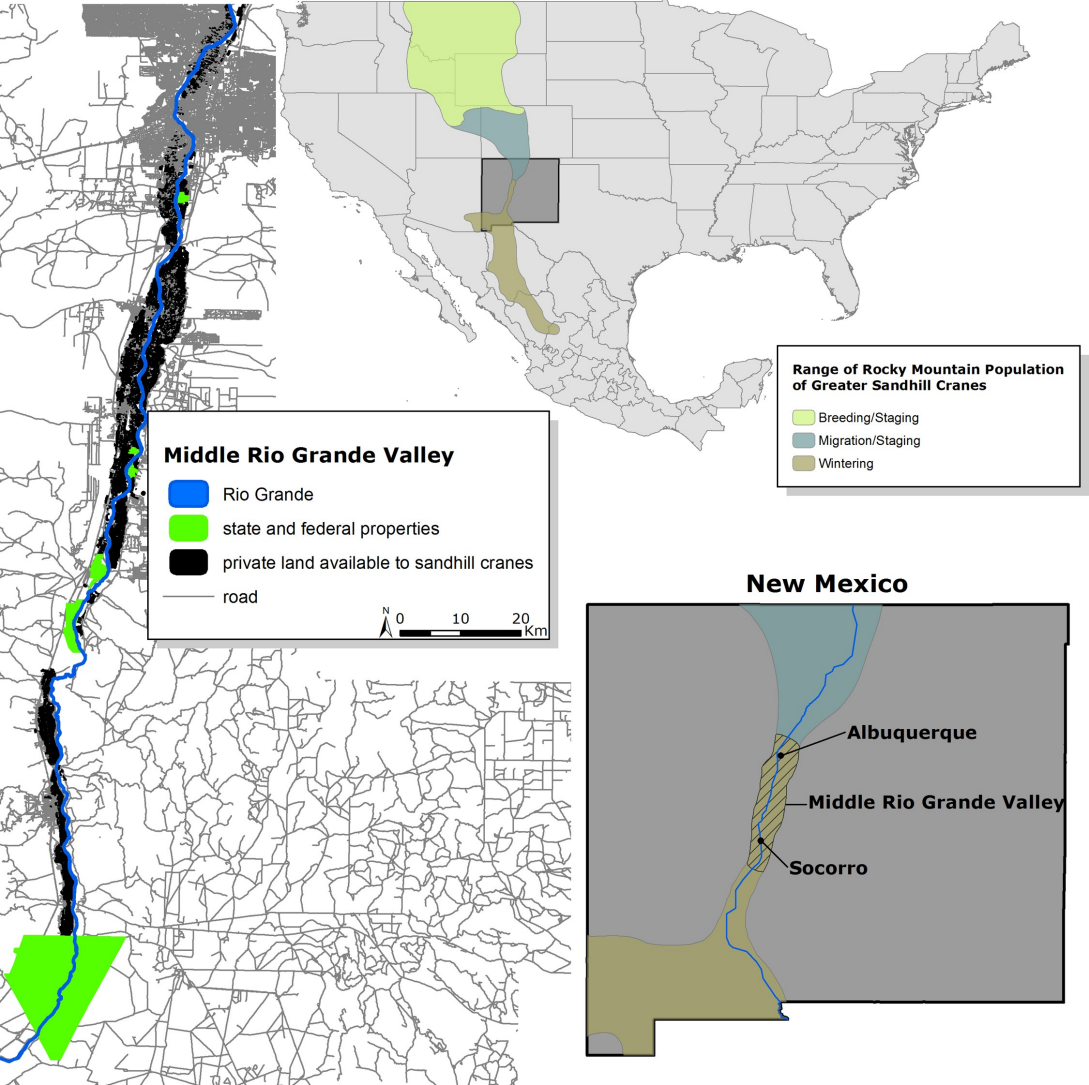
Range of Rocky Mountain Population of greater sandhill cranes, and study area, the Middle Rio Grande Valley of central New Mexico. Green polygons in Middle Rio Grande Valley inset are state (Ladd S. Gordon Waterfowl Complex) and federal (Bosque del Apache and Valle de Oro National Wildlife Refuges) properties on public lands managed to support sandhill cranes during winter.

**Fig 2:**
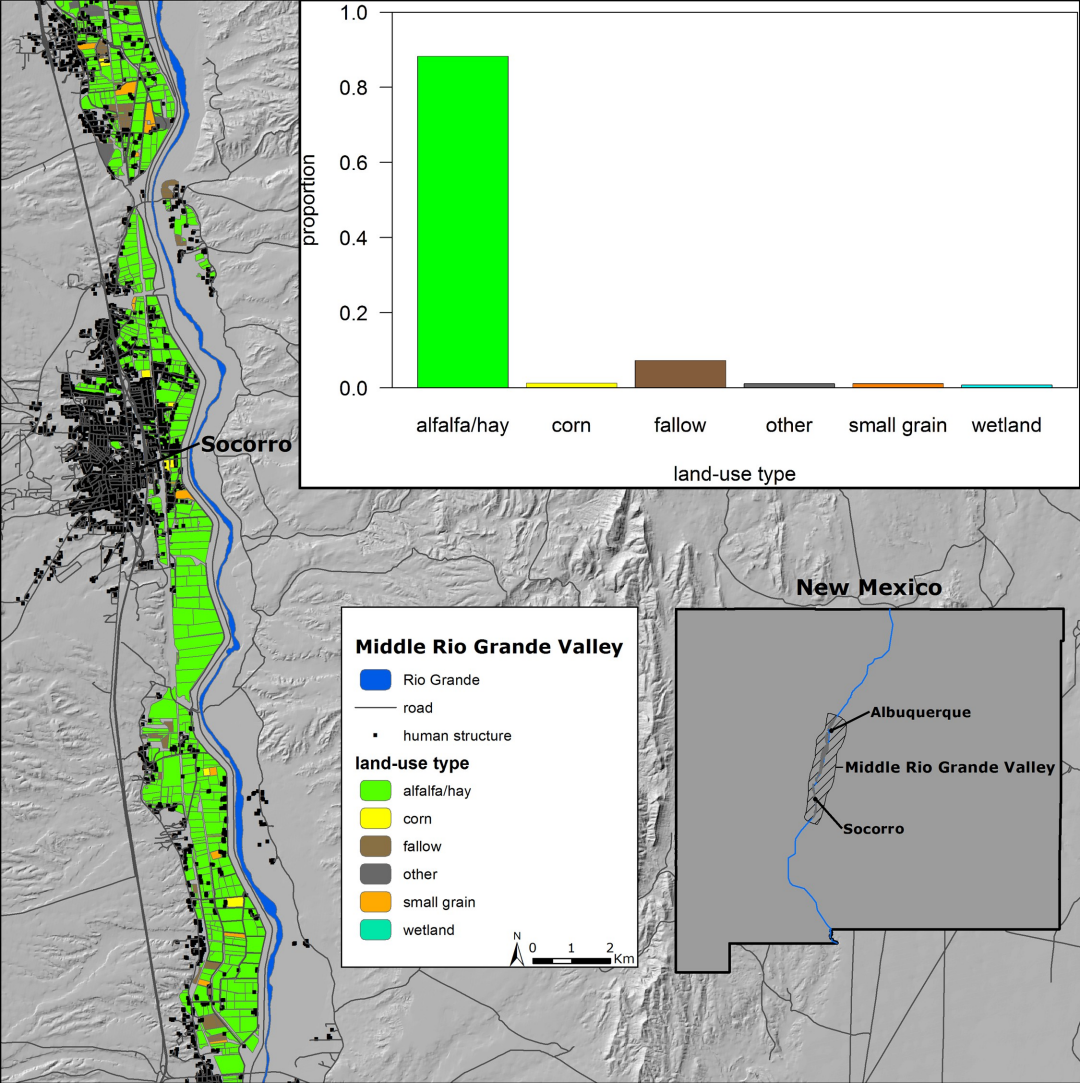
Land-use classification in the Middle Rio Grande Valley of central New Mexico throughout the study period. Alfalfa/hay was the most dominate land-use type on the wintering grounds accounting for over 88% of land-use types classified.

Our study area was located in the MRGV of central New Mexico, USA delimited by the Rio Grande floodplain north of Albuquerque, NM, south to Bosque del Apache National Wildlife Refuge (~200 km; Fig 1). Mean elevation was 1470 m. During winters of 2014–2017, mean temperature was 8°C (range = 7–10°C), and mean precipitation was 19 mm (range = 17–21 mm). Overall conditions were warmer and marginally wetter than long-term trends (GHCN Station ID: USC00291138 and USC00298387, [42]). The Rio Grande riparian corridor is characterized by Rio Grande cottonwood *(Populus deltoides wislizeni*) galleries along with dominant vegetation assemblages including native (coyote willow [*Salix exigua*], Goodding’s willow [*Salix gooddingii*], New Mexico privet [*Forestiera neomexicana]*), and nonnative species (salt cedar [*Tamarisk chinensis*], Russian olive [*Elaeagnus angustifolia*]). The remaining floodplain is a matrix of suburban and urban areas with irrigated agricultural lands [43, 44] consisting primarily of alfalfa (*Medicago sativa*), pastures and grass hay, and some small grain crops (Fig 2). Large numbers of greater sandhill cranes from the Rocky Mountain Population and lesser sandhill cranes (*Antigone canadensis canadensis*) from the Mid Continent Population (collectively ~ 20000 wintering birds), and other migratory birds winter in the MRGV (e.g., light geese [lesser snow geese (*Chen caerulescens)* and Ross’s geese (*Chen rossii*)] and waterfowl). Sandhill cranes are a designated game bird and hunted in the MRGV, however, hunting pressure is very low with hunts restricted to only 12 days during winter, with only a limited number of hunting tags issued for sandhill cranes (draw-based system), a restrictive bag limit (3 sandhill cranes per day), and hunting only permitted on private lands. In addition to the federal property Bosque del Apache National Wildlife Refuge, the Ladd S. Gordon Waterfowl Complex consists of several properties managed by New Mexico Department of Game and Fish (Fig 1) which support migratory birds during winter. These public lands manage wetlands for the benefit of wildlife and administer an agricultural food subsidy program to provide food resources for sandhill cranes and reduce crop depredation on private lands. Specifically, corn (*Zea mays*) is planted during spring to early summer and once mature, standing corn is mechanically manipulated creating a primary food resource for sandhill cranes throughout winter. Publicly managed properties provided the majority of grain crops available to sandhill cranes.

### Capture and satellite transmitter deployment

We used rocket nets to capture sandhill cranes [45, 46] at Bosque del Apache National Wildlife Refuge and Ladd S. Gordon Waterfowl Complex. Once captured, we used plumage characteristics and morphometric measurements to determine age and identify the three subspecies of sandhill cranes (lesser [*Antigone canadensis canadensis*], greater, and Canadian [*Antigone canadensis rowani*]) that winter in the MRGV [47]. For all captured sandhill cranes, we attached a lock-on size nine U.S. Geological Survey (USGS) aluminum band on the left tibia. Additionally, we fitted a subset of captured adult greater sandhill cranes with satellite transmitters (PTT-100 22 g Solar Argos/GPS PTT Microwave Telemetry Inc.). We used a modified leg band with auxiliary markers to attach transmitters to the right tibia of sandhill cranes [48] with both transmitter and USGS aluminum band placed above the tibio-tarsus. Satellite transmitters collected seven GPS fixes daily following these time intervals: 07:00, 08:00, 10:00, 14:00, 16:00, 18:00, and 24:00. Manufacturer reported locational accuracy of satellite transmitters was ± 18 m. We captured and banded a total of 402 sandhill cranes from November to January in 2014–2017 on state and federal properties. We affixed satellite transmitters to 38 adult sandhill cranes captured. From these satellite-tagged sandhill cranes, we acquired 15440 GPS locations. Mean number of GPS locations per sandhill crane was 406 ± 38 (mean ± SE).

### Ethics statement

We acquired all appropriate banding permits from the USGS Bird Banding Laboratory to band and attach transmitters to sandhill cranes (Permit # 23660), and also the necessary U.S. Fish and Wildlife Service and New Mexico Department of Game and Fish permits to capture and study sandhill cranes on Bosque del Apache National Wildlife Refuge and the Ladd S Gordon Waterfowl Complex. Our research protocols were approved by New Mexico State University Institutional of Animal Care and Use Committee (IACUC) and implemented in accordance with institutional guidelines (IACUC # 2014–018).

### GPS locations and defining diurnal and roosting periods

For our study, we were interested in examining spatiotemporal patterns of habitat selection by sandhill cranes during diurnal and roosting periods. The diurnal period is associated with foraging, loafing, and other activities that coincide with daytime and are linked to landscape features used to fulfill these activities such as agricultural fields and wetlands [36]. For the roosting period, we were specifically interested in assessing the function of the Rio Grande as roosting habitat for sandhill cranes. To partition GPS locations into diurnal and roosting periods, we used time intervals that coincided with observed times when most sandhill cranes leave roost sites and begin diurnal activities to when they return to roost sites in the evening. These time periods corresponded to sunrise and sunset. For the diurnal period, we included any GPS fixes that were ≥ 7:00 and < 18:00. For the roosting period, we considered GPS fixes within Rio Grande channel that were ≥ 18:00 and ≤ 7:00. GPS fixes at 7:00 that occurred within the Rio Grande channel were included for the roosting period because these locations were considered representative of individuals that had yet to leave their roost to depart for foraging areas. For both periods, we only considered GPS fixes where altitude data recorded by transmitters did not indicate a sandhill crane was flying. Manufacturer reported altitudinal accuracy of satellite transmitters was ± 22 m. On infrequent occasions, the altitude value was spurious or was not recorded. In these instances, we inspected the location by overlaying the location on high resolution aerial imagery to determine if the sandhill crane was likely flying at the time of the location fix. The overarching objective for each period was to identify important habitat-related features linked to used locations (i.e., GPS locations) and compare these to the suite of conditions available to sandhill cranes.

### Study design for habitat selection analysis

To determine habitat availability, we calculated mean maximum movement distances between successive GPS locations across all sandhill cranes and considered areas within a radius of the mean maximum movement distance from each used location as available habitat. We did not estimate nor determine habitat availability within home ranges (i.e., third order selection [49]) because we were interested in investigating patterns of habitat selection related to each relocation and elucidating factors influencing selection at this more localized and finer scale (i.e., fourth order selection) during diurnal and nocturnal periods, while incorporating spatial heterogeneity of landscape features that might be lost at coarser scales [50]. We did not consider movement distances that were ≥ 10,000 m as these were characteristic of sandhill cranes arriving to, or departing from, the MRGV on fall (November-December) and spring migration (February-March). Following removal of these longer-range movement distances, we identified the maximum movement distance for each sandhill crane, then calculated mean maximum movement distance across all sandhill cranes. We selected mean maximum movement distance because it represented the full movement potential of a sandhill crane once settled on the wintering grounds, excluding migratory movements. Thus, this distance encompassed areas a sandhill crane could select as habitat and avoided underrepresentation of available habitat. When calculating mean maximum movement distance, we only considered distances where time between successive locations was > 3 hr because 3 hr was approximately the average time between our GPS fixes within a 24 hr period, and there is an associated increased uncertainty in movement paths that occurred between locations beyond this time interval. Moreover, mean maximum movement distance for locations ≤ 3 hr apart was only slight higher (6.7 ± 0.29 km[mean ± SE]) compared to locations > 3 hr apart (5.8 ± 0.31 km). Because this difference was marginal, and taking into account the mobility of sandhill cranes, we felt comfortable using mean maximum distance for locations ≤ 3 hr apart because it would have little effect on the patterns of habitat selection inferred from the analysis. Accordingly, habitat availability was considered an area within a radius of 6.7 km from each used location. For each used location, we randomly generated a sample of 50 available locations within this area. Our reasoning for pairing each used location with 50 available locations was twofold. First, simulations have shown that a 1:20 ratio or greater of used to available locations at the higher order scales of habitat selection provides consistent and unbiased parameter estimates in habitat selection analyses [51]. Second, because our radii around each used location were large, we increased the ratio of used to available locations to provide an adequate representation of the variability of conditions within the area available and to permit detection of rarer habitat characteristics [52]. For each used location within the diurnal period, availability was defined as any agricultural field, pasture, or wetland within the 6.7 km radius. Availability for each used roosting location was restricted to any areas within the channel of the Rio Grande.

### Landscape characteristics used for diurnal period analysis

Land use practices are likely the most important human-mediated processes influencing diurnal habitat selection by sandhill cranes [53–56]. Therefore, we sought to understand how current composition and extent of cropping patterns were impacting the distribution and availability of winter habitat. Because an explicit depiction of agricultural patterns was unavailable for the MRGV, we implemented a remote sensing approach to model crop distributions. We first used high resolution (1 m) multispectral aerial imagery acquired in 2014 to digitize agricultural fields, both active (currently farmed) and inactive (fallow or abandoned). Field boundary identification was inclusive of all private and publicly owned lands. We applied the field boundary outputs as a non-uniform sampling grid to support a supervised maximum likelihood model used to predict type and distribution of crops. We used ERDAS Imagine 2013 (Hexagon Geospatial, Norcross, Georgia) to implement the model following methods outlined in [57]. We randomly selected 860 out of 12182 digitized fields and physically surveyed and collected associated crop data in early September 2014. We followed procedures outlined in [58] to determine sampling intensity assuming maximum potential abundance (*P* = 0.5, CI = 95%) of individual crop types surveyed. We randomly selected and withheld 20% of field training data for accuracy assessment. For model training and image classification, we used Landsat 8 Operational Land Imager satellite imagery acquired September 5, 2014 to coincide with timing of field data collection.

We used the model to identify and assign crop values (e.g., corn, alfalfa, sunflower, etc.) to un-surveyed crop fields to produce a spatially continuous and exhaustive estimate of agricultural cropping patterns within the study area. Final classification results were aggregated into five crop classes: alfalfa/hay, corn, fallow, small grains (winter wheat, milo, millet, triticale, and barley), and other to match habitat characteristics for sandhill cranes. The “other” category contained rare crop types that were infrequently detected during sampling (*n* < 9) and were seldom used by sandhill cranes (chili peppers, vineyard grapes, spinach, squash, sunflowers), or were sites identified as abandoned agricultural fields. Overall classification accuracy was 90.9%.

Our modeling results identified a high proportional abundance (88%) of perennial crops (i.e., alfalfa and hay pasture) such that their spatially static nature of occurrence discarded the need to reproduce annual crop distribution estimates. To supplement this assumption, we surveyed the study area each season to identify changes in more dynamic annual crop fields known to be food resources valued by wintering sandhill cranes (i.e., corn [38]). The surveys involved visiting general areas where grain crops were suspected (e.g., dairies), or known (received information from public land managers), to be grown on private lands to confirm their presence, and examining clustering of diurnal GPS locations of sandhill cranes tagged with satellite transmitters to identify and visit agricultural areas that were frequently used and determine crop types of these areas. This procedure ensured documentation of minor landscape changes important in structuring sandhill crane distributions while streamlining the overall analytical demands of the study.

We inventoried land-use types separately for state and federally owned properties managed for wintering sandhill cranes to document spatial distribution of important resources on public lands. Inventories were completed through field visits. Resources identified included agricultural crops as well as wetlands (e.g., managed moist soil impoundments, ephemeral saltgrass meadows). We assigned land ownership with data provided by the U.S. Bureau of Land Management [59]. We used these data to examine habitat selection patterns within ownership and to determine the importance of public and private lands associated with wintering habitat needs of sandhill cranes. We used the spatial distribution and density of human structures (e.g., buildings, domiciles, barns, storage facilities, etc.) as a proxy for anthropogenic disturbance [60]. We digitized all human structures within the study area, then measured distance to nearest human structure for each used and available sandhill crane location. We considered effects of human structure density estimates at three different scales. Around each used and available location, we counted the number of human structures within a radius of 100 m, 500 m, and 1000 m to determine at which scale structure density influenced habitat selection. See below for details on the statistical analysis for habitat selection.

### Landscape characteristics used for roosting period analysis

Previous studies have identified several important geomorphic characteristics of riverine environments that sandhill cranes use for roost sites [61–63].We incorporated these spatiotemporal metrics to describe the Rio Grande within the study area. We first estimated channel width. We used high resolution (1 m) multispectral aerial imagery acquired in 2016 to digitize the active channel and channel centerline. We then used digital transects set perpendicular to the river centerline to calculate width of the active channel at ~30 m intervals. The results provided a continuous estimate of active channel width representative of temporal conditions coincident with location data of sandhill cranes. We next estimated surface water extent within the active channel at bi-monthly intervals throughout the winter (October-November, December-January, and February-March) to account for spatiotemporal shifts in roost site availability. We derived estimates by averaging all available Landsat 8 satellite images occurring within each bi-monthly period and applying a constrained spectral mixture analysis (SMA) [64] to classify areas of water, sandbars, and riparian vegetation (e.g., vegetated islands, vegetated peninsulas, emergent vegetation). All Landsat satellite images were calibrated for atmospheric effects and illumination/viewing geometry [65]. We used the Landsat CFMask band to filter and remove Landsat pixels containing surface anomalies that were negatively influencing model outputs (i.e., cloud, cloud shadow, and snow) [66]. Use of SMA allowed a proportional estimation of each class (i.e., water, sandbars, and vegetation) contained within a 30 m Landsat pixel. This allowed proportional estimations of land-cover features at the sub-pixel level and provided a more detailed estimate of water, sandbar, and vegetation distributions while using the moderate spatial resolution Landsat 8 imagery. Spectral endmember statistics needed to train SMA models were extracted directly from associated Landsat data. We derived riparian vegetation and sandbar endmember statistics from static polygons representing known monotypic examples of these land-cover types. We used an image mask generated from 98th percentile normalized difference water index values (NDWI) [67] to extract spectral endmembers for water. We calculated a NDWI mask for each bi-monthly period. Mask extents were coincident to large deep water reservoirs proximal to the study area. We considered proportional estimations of land-cover classes within individual pixels derived from SMA to infer geomorphic characteristics of the active channel within each bi-monthly period. For example, if the proportion of water in a pixel was 0.9, that area of the Rio Grande was completely water, and depending on channel width, likely deeper water as little sandbar signature was present. If, for example, the water proportion of a pixel was 0.4 and sandbar proportion was 0.6, that pixel was characterized by emergent sandbars with shallow water present. All bi-monthly models were spatially representative of proportion water, sandbars, and riparian vegetation occurring within a 30 m grid coinciding with each used and available location during the roosting period. We repeated all procedures for each year of the study (2014–2017) and used Google Earth Engine, a cloud-based geospatial processing platform, to derive the image classification procedures [68].

Riverbank vegetation height has been identified as an important characteristic of riverine environments influencing roost site selection by sandhilll cranes [63]. We incorporated this variable into our modeling framework by using available Light Detection and Ranging (LIDAR) data to estimate riparian vegetation height. LIDAR data was collected by fixed-wing aircraft in 2012. We processed LIDAR point-cloud and filtered first-return results to create a gridded 10 m surface representing vegetation top of canopy elevations. We then subtracted the top of the canopy surface from a 10 m bare earth digital elevation model to estimate riparian vegetation canopy height. Height estimates were constrained to 10 m grid cells occurring within 50 m of the Rio Grande’s active channel to allow comparisons to, and remain consistent with, methods outlined by [63] where roost site selection of sandhill cranes was studied along the Platte River in Nebraska, USA. We estimated mean vegetation height on channel banks within 100 m, 500 m, and 1000 m stretches of the river surrounding each used and available location during the roosting period to examine influence of this metric across multiple scales. We considered impacts of human disturbance during the roosting period and used known factors (e.g., distance to bridge and human structure) that influence roost site selection [61, 63, 69]. Distance measurements were made between used and available roosting locations and the nearest human structure and bridge. Density of human structures within a 1000 m radius of used and available locations were also considered as an influence on roost site selection. We chose a 1000 m radius because at any smaller scale, very few human structures were present due to the large riparian areas flanking each side of the Rio Grande. All geospatial analyses other than those performed in Google Earth Engine and ERDAS Imagine were done in ArcGIS 10.5.1 (ESRI, Redlands, California, USA).

### Statistical analysis

To evaluated habitat selection and compare characteristics between used and available locations, we used multimodel inference from conditional logistic regression with generalized estimating equations, which is a preferred approach for case-control designs with longitudinal data [70]. This approach accounts for misspecification of correlation structures and generates robust standard errors by using independent clusters to account for non-independence among observations, where observations within one cluster are considered statistically independent of another cluster [70, 71]. To fit models, we used the R package *survival* [72]. Simulations have shown that ≥ 20 clusters are required to avoid biased parameter estimation, and that assignment of one individual to a cluster results in higher precision in estimates when ≥ 30 individual animals are in the sample [70]. Since we tagged and monitored over 30 individual sandhill cranes through the study, we assigned each individual to its own cluster.

We developed a candidate set of models that we hypothesized would likely influence patterns of diurnal and roosting habitat selection by sandhill cranes (specification, justification, and model selection results for candidate set of models detailed in S1 Appendix). We used variance inflation factors (VIF) to assess multicollinearity among covariates in all models. If the VIF was ≥ 5 for any covariate in a model, the covariate was not included in the model [73,74]. To compare models, we used Quasi-likelihood under Independence Criterion (QIC) [75]. Similar to other information criterion, QIC is aimed at striking a balance between model complexity and explanatory power, and it was most appropriate for our applications because QIC is deigned to evaluate models where the full likelihood is not defined and accounts for within-individual autocorrelation [75]. We did not model average because our model sets were not orthogonal, we included interaction terms which changes the interpretation of main effects, and model-averaged estimates can be unreliable when correlations exist among covariates [76].

We used *k*-fold cross validation under case-control study designs to evaluate predictive accuracy of the most supported and parsimonious model [77–79]. We followed the methods outlined in [78]. We partitioned the dataset, while keeping strata intact, and randomly selected 80% of strata for training the model and the other withheld 20% for validation. For each stratum, the predicted value for a used location was ranked against the available locations, then rankings were tallied into bins with number of bins equal to the number of records in each strata. Then we used Spearman rank correlations ${(\overset{-}{r}}_{s})$ to compare bin ranking and the frequencies of predicted values of used locations within each bin. We repeated this process 1000 times, then calculated ${\overset{-}{r}}_{s}$ across all iterations. Models that had strong predictive performance have ${\overset{-}{r}}_{s}$ values closer to 1. We used the R package *hab* [80] to perform the cross validation. We used the most supported and parsimonious model (highest QIC value) from each candidate model set to draw inference and predict relative probability of use throughout the study area to delineate distribution of diurnal habitat and roosting habitat (we provide an example of a bimonthly interval for one of the study years) for sandhill cranes during winter and identify areas of importance. All statistical analyses were done in Program R [81].

### Behavioral observations

Although GPS fixes and habitat selection analysis provide insight into sandhill crane behavior, such location-based proxies do not guarantee detection of nuanced behaviors that are associated with what may be important habitat characteristics. To expand our understanding of behavior of sandhill cranes, we observed diurnal behavior of sandhill cranes on state and federal properties during winter. We used focal sampling at discrete intervals [82] to observe diurnal behaviors of individual sandhill cranes from sunrise to sunset. For each sampling occasion, an observer was assigned a set of land-use types on federal and state properties each corresponding to a time of day (07:00–18:00) and observed behavior of individual sandhill cranes in the assigned land-use type at the assigned time of day. We identified alfalfa fields, corn fields, fallow fields, moist-soil managed wetlands as available land-use types because they were the most dominant on the state and federal properties and used by sandhill cranes throughout the diurnal period. Upon arrival to the assigned land-use type, the observer would locate a group of sandhill cranes with a spotting scope (Vortex 20-60x80 mm), look away and randomly move the spotting scope horizontally and vertically, reacquire the group of sandhill cranes and select the sandhill crane nearest to or at the center of the field of view of the objective lens. Once a sandhill crane was selected, the observer recorded the behavior of the sandhill crane every 10 sec within a 30 min interval. Observations were made at distance as to not disturb or influence behavior (generally ≥ 200 m). Categories of associated behaviors were: comfort, foraging, locomotion, resting, and social. If the observer lost sight of the sandhill crane for more than five minutes at any point during the 30 min observation period, or if the sandhill crane permanently flew out of view, the observer would randomly select a new individual to observe and reinitiate the protocol. Behavioral data from partial observations that did not span the full 30 min period were included in the analysis. We did not observe sandhill crane behavior during the roosting period because previous studies have shown that sandhill cranes only engage in resting behavior while roosting [83]. We used behavioral observations to gain further insight into models that best predicted diurnal habitat selection by sandhill cranes and determine how spatial and temporal variability in specific behaviors might be important when considering habitat needs of the population. We observed behaviors of 678 sandhill cranes from November to February in 2014–2017. Estimated number of observations per sandhill crane was 142 ± 58 (Mean ± SD).

## Results

### Diurnal habitat selection

**Fig 3:**
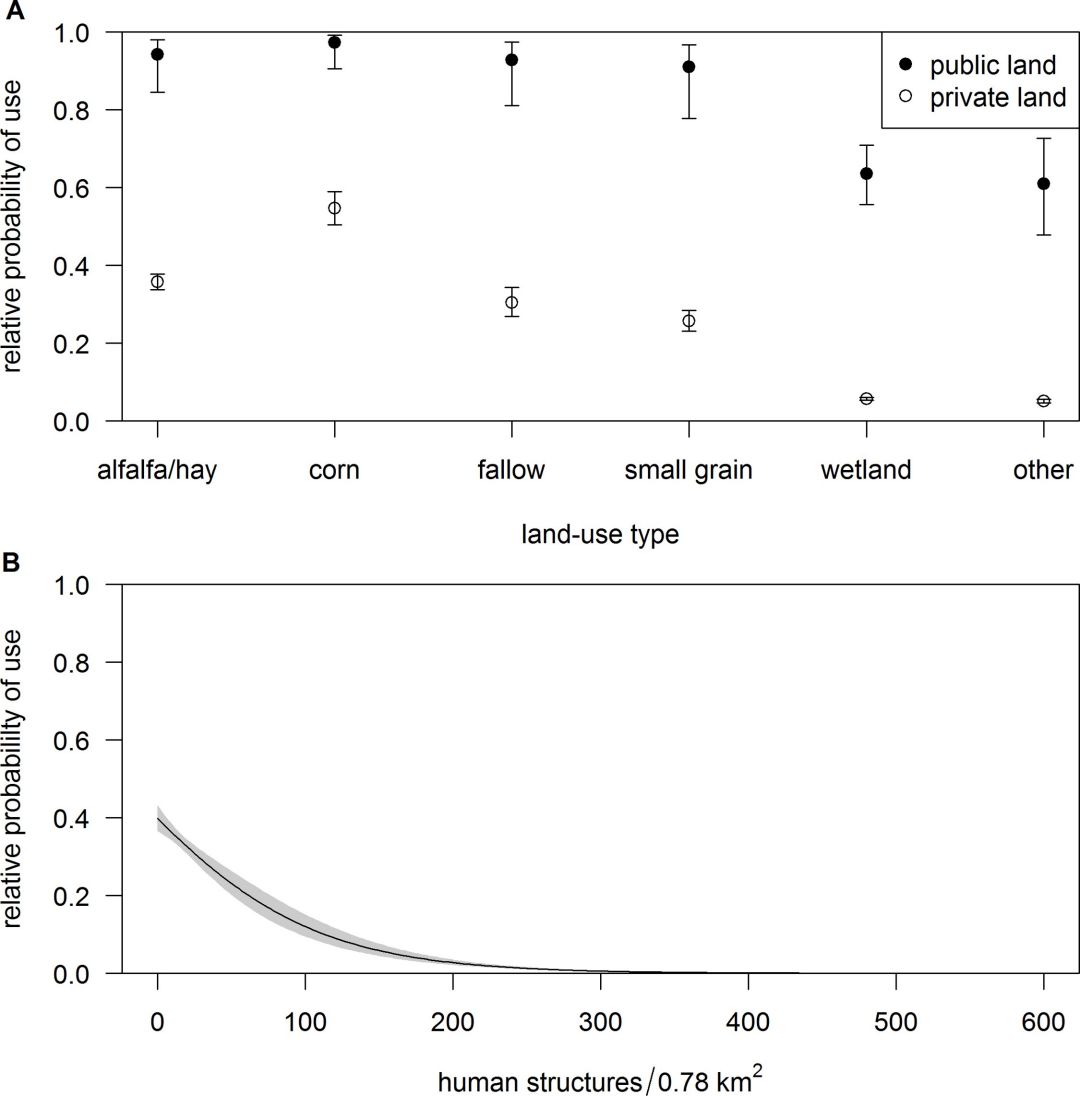
Patterns of diurnal habitat selection by sandhill cranes in the Middle Rio Grande Valley of New Mexico. Diurnal habitat selection by sandhill cranes during winter was best described by relative probability ± 95% confidence interval of using (A) different land-use types on public and private properties and (B) relative probability of use ± 95% confidence interval in relation to density of human structures within a distance of 500 m.

The predominant land-use type classified throughout the study period was alfalfa/hay, which accounted for 88% of all land-use types classified (Fig 2). Model comparison results for diurnal habitat selection revealed the most supported model in the candidate set, carrying nearly all of the QIC weight, contained the additive effects of land-use type, land ownership (public or private) and density of human structures within a 500 m radius (Table A and Table B in S1 Appendix and Table 1). Sandhill cranes had a higher probability of selecting all land-use types on public lands compared to private lands (Fig 3). Land-use types with high relative probability of use on public lands included alfalfa fields, corn fields, fallow fields, and small grain fields. On private lands, corn fields had the highest relative probability of use (Fig 3A). Furthermore, relative probability of use decreased as density of human structures within a 500 m radius increased (Fig 3B). *K*-fold cross validation of our most supported model predicting diurnal habitat selection by sandhill cranes indicated high predictive power with a ${\overset{-}{r}}_{s}$ of 0.86 (range = 0.74–0.92).

**Table 1:** Parameter estimates and robust standard errors of most supported model describing diurnal habitat selection by sandhill cranes on their primary wintering area, the Middle Rio Grande Valley of central New Mexico.

Parameter	Estimate	SE	*P*
land-use type^a^			
corn	0.773	0.070	< 0.001
fallow	-0.240	0.157	0.126
small grain	-0.478	0.130	< 0.001
wetland	-2.230	0.158	< 0.001
other	-2.342	0.197	< 0.001
land ownership^b^	3.373	0.165	< 0.001
structure density_500m ^c^	-0.377	0.102	< 0.001

^a^land-use type included alfalfa fields, corn fields, fallow fields, small grain fields, wetlands, and an other category (see text for description). The reference category was alfalfa fields.

^b^ land ownership = Public or private land ownership. Reference category was private land ownership.

^c^ structure density_500m = density of human structures within a 500 m distance from used and available locations.

### Roosting habitat selection

**Fig 4:**
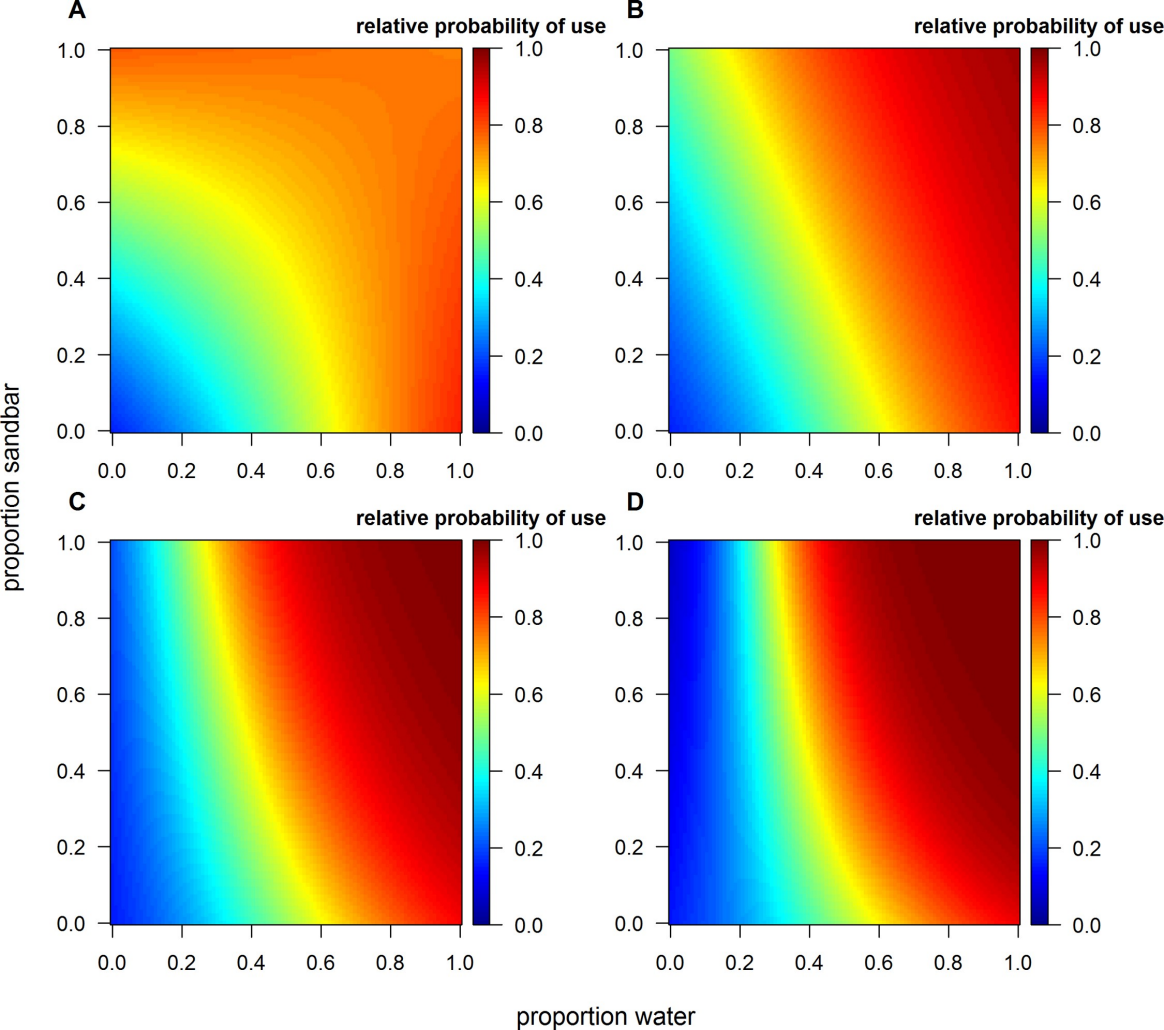
The interplay among morphological characteristics of the Rio Grande considerably influenced relative probability of roost site selection by sandhill cranes during winter in the Middle Rio Grande Valley of central New Mexico. Relative probability of using areas within the Rio Grande for roosting increased as proportion of water and sandbars increased with increasing channel width as exemplified by (A) 50 m width, (B) 100 m width, (C) 150 m width, and (D) 200 m width.

**Fig 5:**
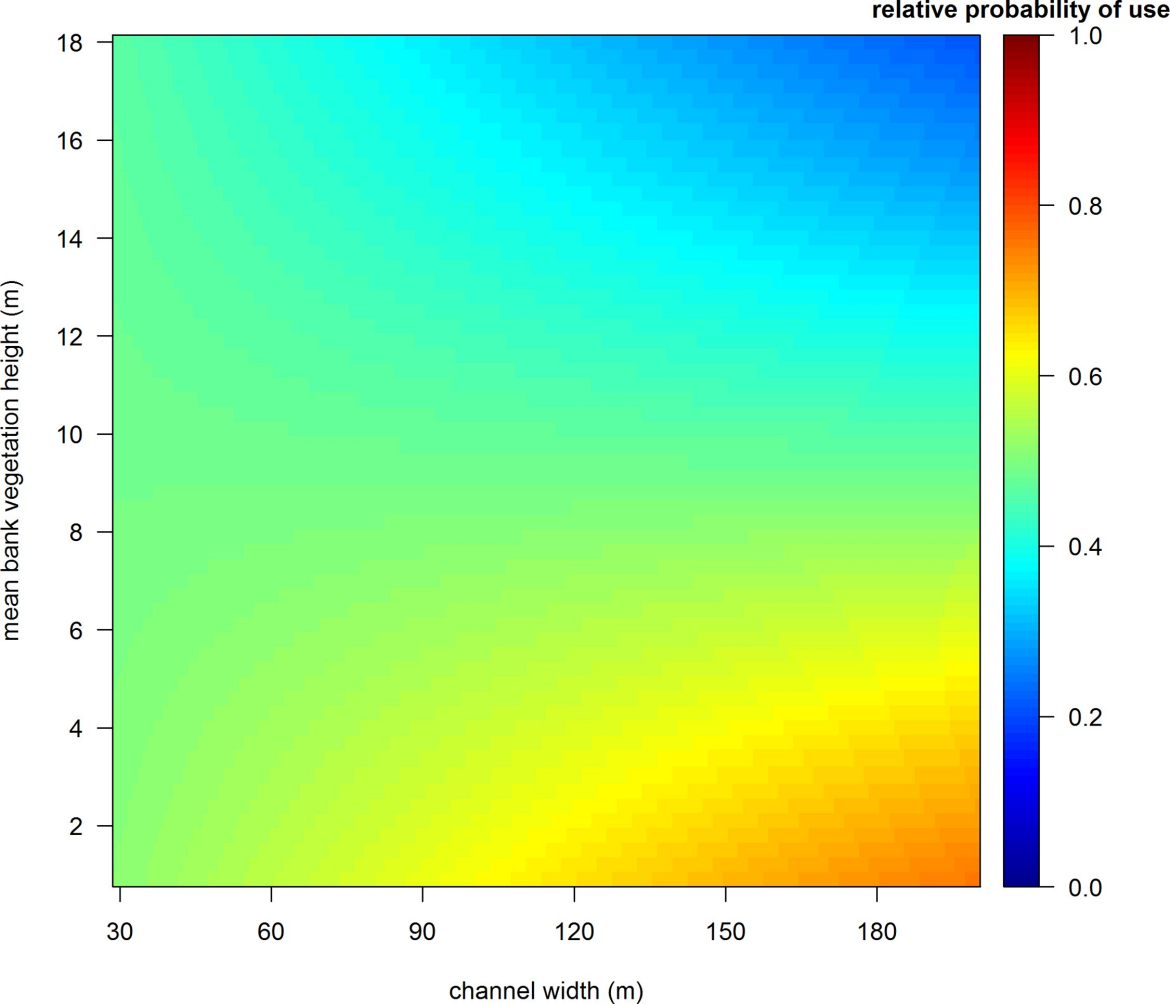
Roost site selection in the Rio Grande by sandhill cranes wintering in the Middle Rio Grande Valley of central New Mexico was influenced by the interactive effect of channel width and mean bank vegetation height. In general, relative probability of use increased as channel width widened and mean bank vegetation height decreased.

**Fig 6:**
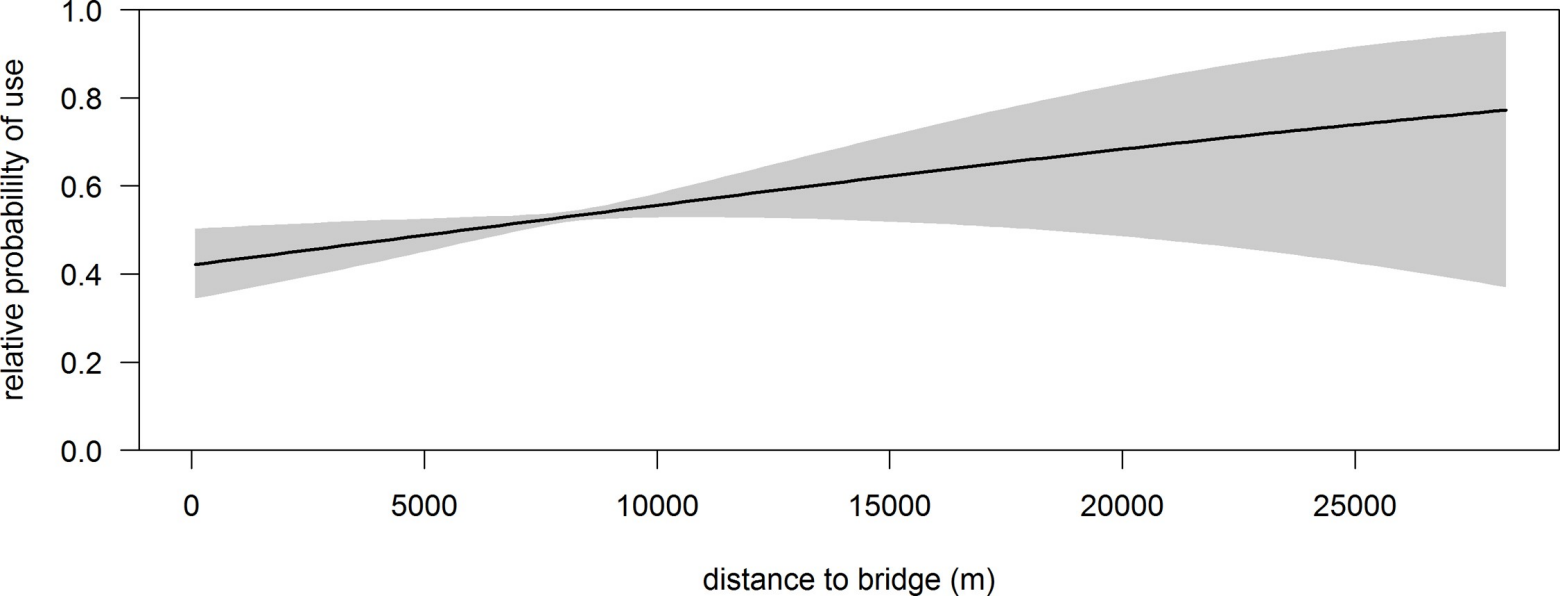
In the Middle Rio Grande Valley of central New Mexico, predicted relative probability of a sandhill crane roosting in the Rio Grande during winter decreased as proximity to bridges increased. Shaded region is 95% confidence interval.

During the roosting period, the two most supported models describing roosting habitat selection by sandhill cranes included a three-way interaction between channel width, proportion of water, and proportion of sandbars, and an interaction between channel width and mean bank vegetation height within a 500 m stretch of the Rio Grande (Table C and Table D in S1 Appendix). Of these two, the model carrying the highest model weight (82%) included the additional effect of distance to bridge (Table C and Table D in S1 Appendix and Table 2). We decided to use this model (Table 2) to make inference for roosting habitat selection by sandhill cranes during winter because it was well supported, had the highest explanatory power, and included an important covariate that was a proxy for anthropogenic disturbance. Furthermore, *k*-fold cross validation demonstrated this model had high predictive accuracy (${\overset{-}{r}}_{s}$ = 0.94, range 0.88–0.97). The three-way interaction between channel width, proportion of water, and proportion of sandbars increased the relative probability of selecting areas within the Rio Grande as the channel widened and contained a higher mixture of water and sandbars, indicative of a more braided channel system morphology (Fig 4). Additionally, the interaction between channel width and mean bank vegetation height influenced roosting habitat selection. A narrower channel width, which is more incised and has reduced lateral flows, supports more woody vegetation [15] and in turn can have higher mean bank vegetation heights which decreased relative probability of use (Fig 5). Relative probability of using a location within the Rio Grande for roosting also increased as distance from bridges increased (Fig 6).

**Table 2:** Parameter estimates and robust standard errors of most supported model describing roosting habitat selection by sandhill cranes in the Rio Grande on their primary wintering area, the Middle Rio Grande Valley of central New Mexico.

Parameter	Estimate	SE	*P*
channel width	0.060	0.059	0.312
sandbar	0.207	0.095	0.029
water	1.404	0.044	< 0.001
bank veg height	-0.314	0.116	0.007
distance_bridge_	0.454	0.201	0.024
channel width × sandbar	0.016	0.045	0.726
channel width × water	0.248	0.041	< 0.001
sandbar × water	0.114	0.077	0.140
channel width× bank veg height	-0.117	0.061	0.054
channel width × sandbar × water	0.133	0.054	0.013

channel width = width of Rio Grande channel

water = proportion of water within 30 m pixel of channel

sandbar = proportion of sandbars within 30 m pixel of channel

bank veg height = mean height of channel bank vegetation within a 500 m distance surrounding used/available locations

distance_bridge_ = proximity to nearest bridge

### Behavior observations

**Fig 7:**
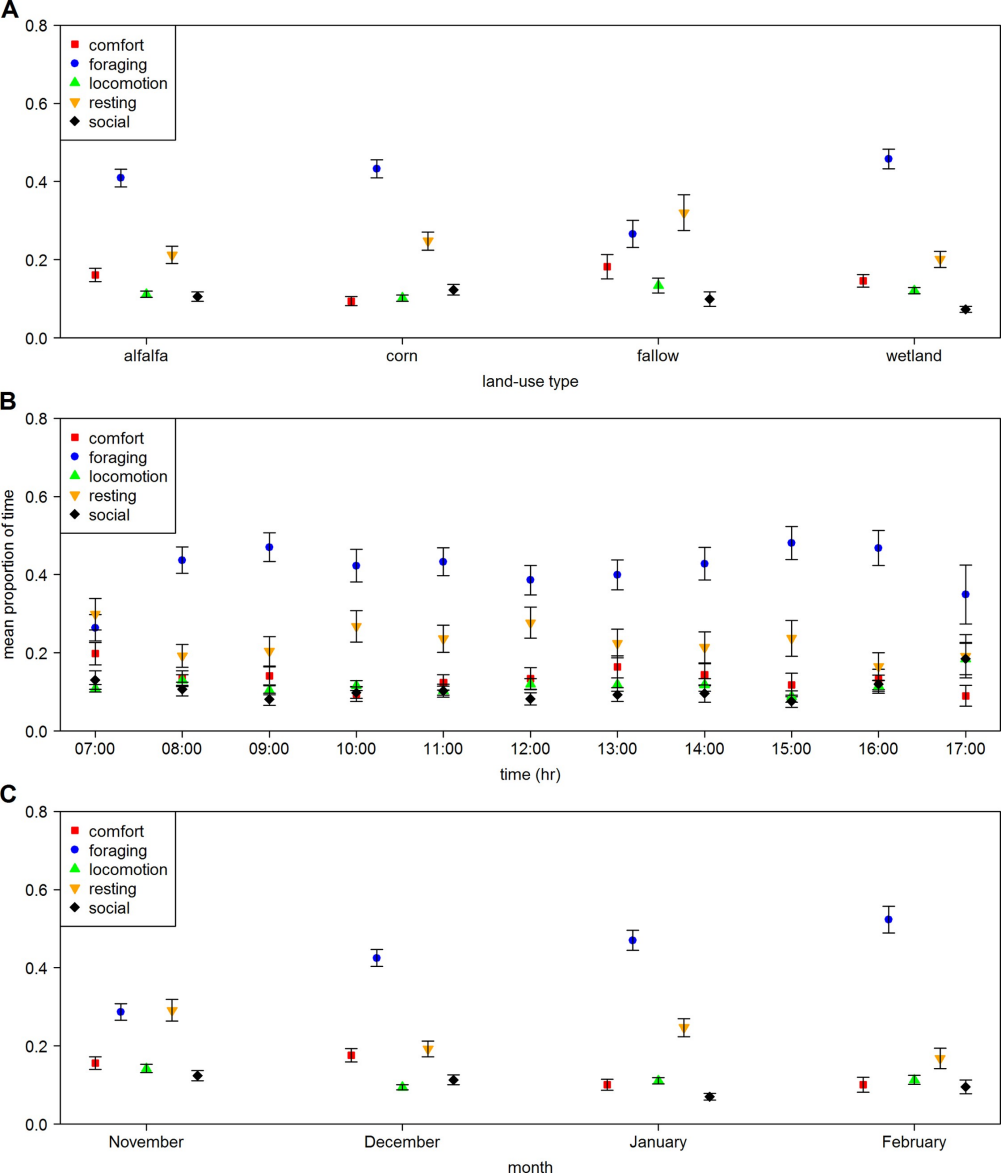
Mean proportion ± SE of time spent engaging in different behaviors by sandhill cranes during winter in the Middle Rio Grande Valley of central New Mexico by (A) land-use type, (B) time of day, and (C) month of winter.

Sandhill cranes predominately engaged in foraging and resting throughout winter (Fig 7). Overall, behaviors did not vary considerably by land-use type (Fig 7A), however, sandhill cranes spent the highest proportion of time foraging in alfalfa fields, corn fields, and wetlands, and the highest proportion of time resting in fallow fields. Foraging was the dominant activity during the day and appeared to be slightly bimodal, with sandhill cranes reducing foraging and increasing time spent resting at sunrise (7:00), midday (12:00), and sunset (17:00) (Fig 7B). Across the months that spanned winter, from arrival to wintering grounds in November during fall migration, and up to departure on spring migration in February, time spent foraging by sandhill cranes increased, suggesting energetic preparation for spring migration (Fig 7C).

## Discussion

### Diurnal habitat selection

During the diurnal period, sandhill cranes demonstrated avoidance of areas with high density of human structures (Fig 3B). As the human footprint increases in areas on the wintering grounds, sandhill cranes will likely avert use of these areas for foraging and loafing because impacts of increased levels of disturbance outweigh potential resource benefits these areas may offer; an avoidance response that has been shown in other populations [54, 84, 85]. Land ownership was equal if not a more important factor influencing sandhill cranes, that showed strong selection towards public versus private lands (Fig 3A). Federal and state properties that are managed for sandhill cranes throughout winter, compose a small fraction of the MRGV (~ 0.07% of land area), yet accommodate most of the wintering sandhill cranes. During the diurnal period, for example, 75% of locations of sandhill cranes tagged with satellite transmitters occurred on these managed public lands. The agricultural food subsidy program, and wetland management that emulates historical wetland conditions on public lands, effectively provide sandhill cranes with important foraging and loafing habitat during winter. Although land-use types such as corn fields on private lands were moderately important, harvest practices (i.e., silaging prior to sandhill crane arrival) likely reduce grain availability to sandhill cranes resulting in lower relative probability of use. Current agriculture practices on private lands in the MRGV, now dominated by alfalfa and pasture (Fig 2) valued for their high economic yield and demand for livestock forage, further underscore the critical role of managed public lands in sustaining wintering populations.

Closer examination during the diurnal period suggested that behavior of sandhill cranes on public land was fairly consistent across land-use type (Fig 7A) with foraging and resting (loafing or sleeping) accounting for the highest proportions of behaviors. All land-use types, with the exception of reduced foraging rates observed in fallow fields, supported both of these behaviors suggesting sandhill cranes used land-use types on managed properties similarly during the diurnal period. Resources within each of these land-use types, however, likely fulfill different dietary requirements. Corn, for example, contributes proportionally the most to sandhill crane diets [38] and provides a highly metabolizable carbohydrate [86] used to maintain and store energy reserves [54]. Managed moist-soil wetlands are flooded seasonally and also contain nutritionally valuable plant-based resources (e.g., alkali bulrush [*Bolboschoenus maritimus*], yellow nutsedge [*Cyperus esculentus*], [37, 38]), as well as provide a source of protein via invertebrate communities [87]. Together these land-use types supply sandhill cranes with forage resources and areas with minimal disturbance to carry out diurnal activities while conserving energy. Foraging peaked in mid-to-late morning and mid-to-late afternoon with proportion of time spent resting highest in early morning, midday, and evening (Fig 7B); a foraging cycle resembling other studies investigating Gruid spp. behavior [88, 89]. The proportion of time spent foraging increased throughout winter, leading up to spring migration in mid-February (Fig 7C). Ramping up foraging (i.e., hyperphagia) prior to an energetically expensive event, such as migration, is a common strategy in migratory birds [90, 91], and reiterates the importance of public lands and land managers synchronizing food resources with timing of highest population needs [38], and providing sandhill cranes with a consistent resource base until spring departure.

### Roosting habitat selection

Historically, the Rio Grande was a highly dynamic river system marked by frequent pulses of flooding within the floodplain creating a wide and braided river channel interspersed with sandbars, wetlands, and wet meadows [15, 92], an ideal collection of high quality habitat features for sandhill cranes and many other wildlife. Presently, however, the historic flow regime of the Rio Grande has been substantially altered, creating narrower and incised channel morphology, and sedimentation loads that have allowed establishment of invasive woody vegetation such as Tamarisk [15, 93]. Much of this change owing to human alteration of the natural hydrology, a deeper water table, and regional drought [94], has eliminated important riverine habitat once used by sandhill cranes [92].

Channel width played an important role in selection of roost sites for sandhill cranes, with relative probability of use increasing at sites with a wider channel and higher occurrence of both sandbars and water (Fig 4), and in areas with low bank vegetation (Fig 5). Our results suggest that an incised river channel with deeper water and limited lateral flow, characteristics of many reaches of the Rio Grande, does not provide high quality roosting habitat. Moreover, these characteristics promote establishment of woody vegetation because periodic flooding of the overbanks rarely occurs [44], further decreasing suitability of these areas as roost sites. In other studies, avoidance of areas with tall bank vegetation by sandhill cranes has been attributed to anti-predator behavior, where perceived predation risk in these areas might be heightened because of obstructions to detect predators [63, 69]. Although we do not have evidence to support this postulation, our results suggest the general qualities of areas with a narrower channel in the Rio Grande are avoided by sandhill cranes. Moreover, so are areas near bridges, which can have a large disturbance effect on roosting sandhill cranes [63, 95]. Finally, we did not find an effect of distance between areas with high relative probability of use during the diurnal period and roosting locations, but the energetic importance of this relationship should not be discounted. Longer flight distances between foraging areas and roost sites can influence habitat selection and have negative physiological effects for birds [53, 96, 97], hence should be included as a consideration for management of species where foraging and roosting habitat are separated in space.

Outside of the Rio Grande, publicly managed wetlands provided alternative roost sites that may become increasingly important if degradation of riverine habitat increases. Conversely, if water rights needed to manage public wetlands are jeopardized or drought creates shortages in allocated water supplies, the ability of natural resource agencies to provide roosting habitat may be restricted [39]. Reliance of sandhill cranes on public wetlands warrants further evaluation to determine how changes to water use policy may affect resources. Predictions of increasing water scarcity could have considerable influence on distribution and availability of habitat for sandhill cranes.

### Predicting habitat selection on the wintering grounds

**Fig 8:**
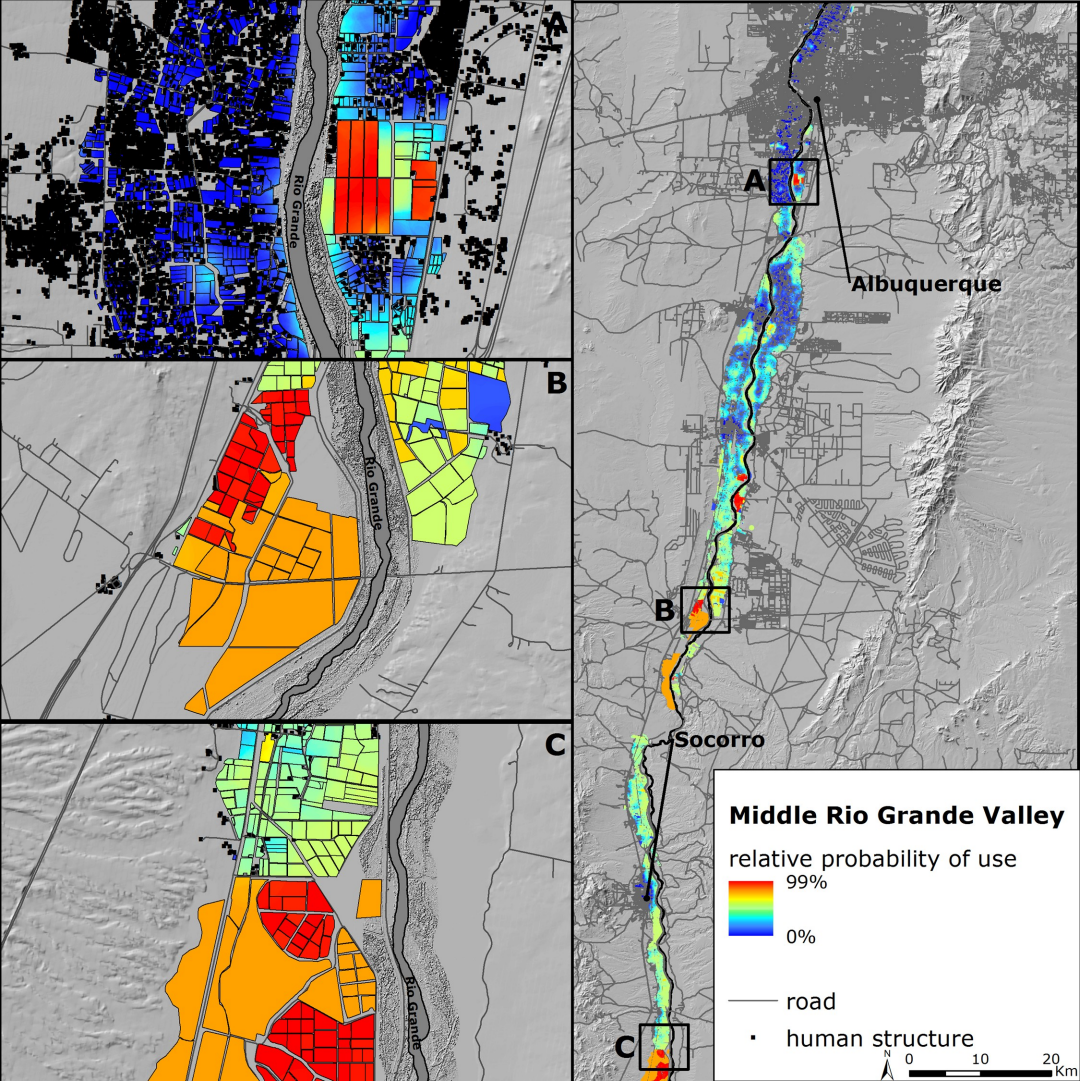
Relative probability of use by sandhill cranes during the diurnal period in the Middle Rio Grande Valley of central New Mexico predicted from the most parsimonious model. The three insets are zoomed in perspectives of several high relative probability of use state and federal properties, (A) Valle de Oro National Wildlife Refuge, (B) Bernardo Waterfowl Management Area operated by New Mexico Department of Game and Fish, and (C) the northern portion of Bosque del Apache National Wildlife Refuge.

**Fig 9:**
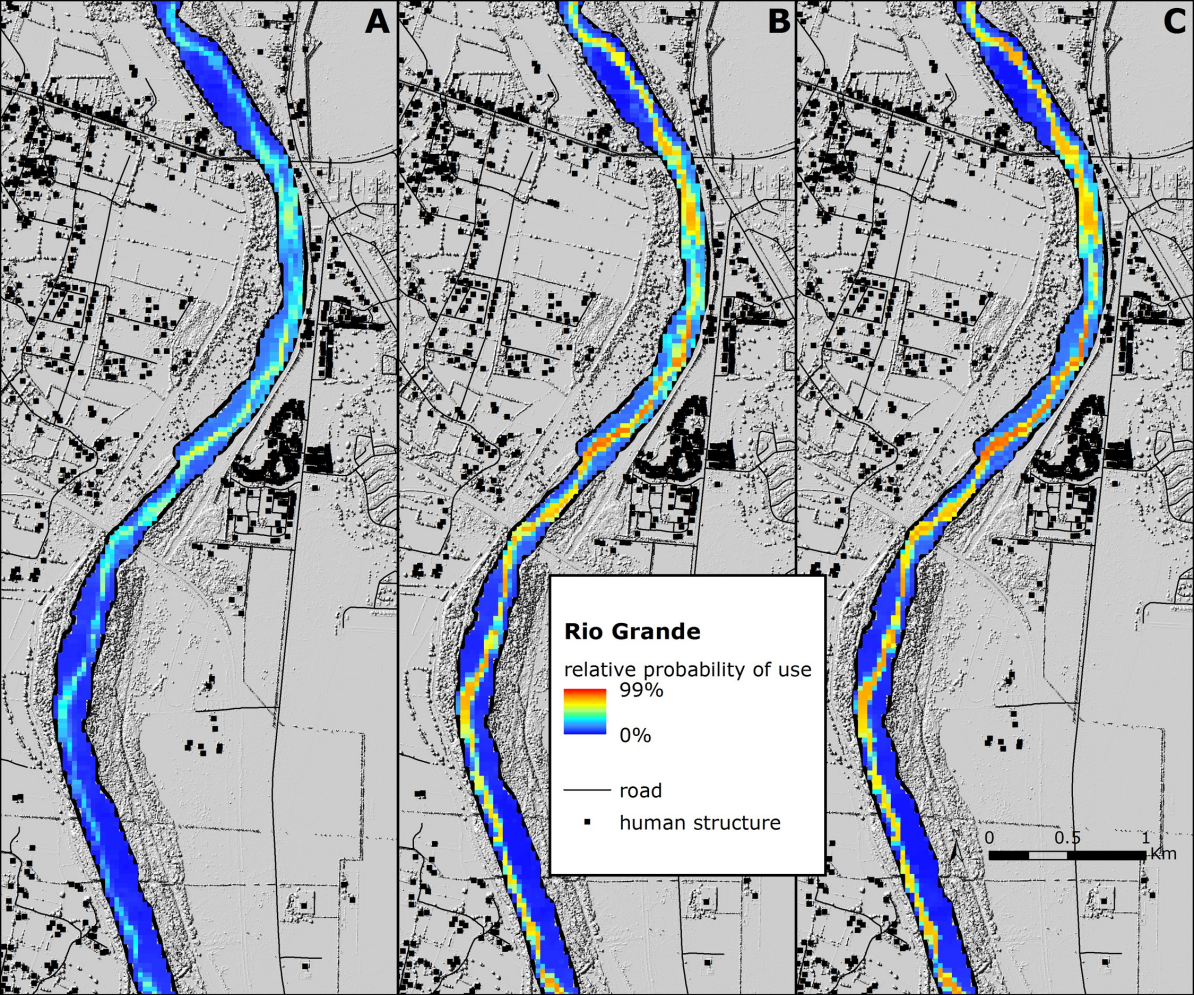
Relative probability of use by sandhill cranes during the roosting period in the Middle Rio Grande Valley of central New Mexico predicted from the most parsimonious model. The three panels are predictions for the same stretch of the Rio Grande in 2016–2017 for USGS Landsat 8 imagery acquired in (A) October and November, (B) December and January, and (C) February and March.

Existing land-use practices on private lands and associated disturbances likely limit the extent and quality of habitat on the wintering grounds. Heavily reliant on natural resource agencies that manage public lands for wintering migratory birds, and the existing riverine conditions that support roosting, the MRGV remains a critical wintering area for sandhill cranes but not without risk. Predicted relative probability of use throughout the MRGV accentuates the importance of federal and state properties but also reveals decreased relative probability of sandhill cranes using private resources, particularly near human developments (Fig 8). More dynamic, predicted relative probability of roost site occurrence in the Rio Grande is dependent on fluctuations in water depth and physical characteristics (e.g., vegetation height) which influence the relative probability of where sandhill cranes may roost within the same stretch of river but at different periods throughout winter (Fig 9). The ability to simulate near real-time conditions of the Rio Grande as it pertains to roosting enabled us to correctly represent unbiased patterns of roost site selection and control for within-season changes that influenced availability. Collectively, our results provide a benchmark for a moving target that can help direct management strategies for wintering sandhill cranes and support their persistence in a future marked by uncertainty.

### Securing winter habitat in the future

Long-term land use and riverine trends in the MRGV have likely increasingly isolated wintering habitat for sandhill cranes on managed public lands. State and federal properties that support sandhill cranes and other migratory birds, however, contribute only a small proportion to the total land area in their winter range (Fig 1). Although these properties have a proven track record of success in both the ecological and socioeconomic dimensions (e.g., reducing crop depredations on private lands), considering a shared public-private lands strategy may be necessary to expand and distribute habitat-related resources through volunteer incentive-based programs (e.g., USDA NRCS Farm Bill). Private lands conservation supportive of migratory bird needs can provide an atonable solution addressing both agricultural and wildlife sustainability. Alternatively, recreational value of public lands used by migratory birds may generate leverage that make acquisition of additional properties financially and biologically justifiable, but may be challenging given the budgetary climate of natural resource agencies. Growth of public conservation would increase recreational opportunities in the form of hunting and ecotourism (e.g., birdwatching and wildlife photography) that can have a positive economic impact on local economies [98].

Maintenance and improvement of roosting habitat in the Rio Grande will require continued dialogue to address complex water use demands in combination with restorative actions to reduce nonnative woody vegetation impacting riparian habitat. Many species can benefit from such restoration efforts in the Rio Grande [16, 18]. Conflicts over water in arid and semi-arid environments are increasingly exacerbated by more frequent drought and warming temperatures [99, 100]. Such drivers of environmental change will undoubtedly influence future circumstances that must be matched with commensurate shifts in land, river, and wildlife management practices to meet conservation objectives for wildlife populations and their habitats.

## Supporting information

S1 Appendix Candidate model set development and model selection results for conditional logistic regression models used for analysis of habitat selection by sandhill cranes. (DOCX)

S1 Data Data used for analysis of habitat selection by sandhill cranes. (ZIP)
