# Supplementary material for: Land Use, anthropogenic disturbance, and riverine features drive patterns of habitat selection by a wintering waterbird in a semi-arid environment
Source: PLoS One. 2018 Nov 7;13(11):e0206222. doi: 10.1371/journal.pone.0206222 (PMC6221299; doi:10.1371/journal.pone.0206222)
Supplement: S1 Appendix — (DOCX) [file pone.0206222.s001.docx]

## S1 Appendix: Candidate models and model selection results to determine habitat selection during the diurnal period and roosting period

### Diurnal period

We developed a candidate set of models that we hypothesized would likely influence patterns of diurnal habitat selection by sandhill cranes in the Middle Rio Grande Valley of central New Mexico, USA during winter (Table A). The primary factors we considered were land use and land ownership (i.e., public versus private) and these variables were included in most of the models. Additionally, we considered how density of human structures within different distances (100 m, 500 m, and 1000 m) from used and available locations might alter habitat selection. Several variables had high collinearity (VIF > 5) with other variables and were not included in the model set. These included distance to human structures, and inclusion of density of human structures as an interaction term with other covariates. We hypothesized that land-use type is likely the primary driver influencing habitat selection and diurnal behavior of sandhill cranes on the wintering grounds (Table A model 1). Alternatively, we hypothesized that public lands on the wintering grounds, which include federal wildlife refuges and state waterfowl management areas, provide all habitat requirements needed to support sandhill cranes (Table A model 2). Furthermore, diurnal habitat selection might not only be driven by how different land-use types fulfill diurnal habitat needs since the magnitude of selection of land-use types might also vary on public versus private land (Table A model 3). We also considered several models that captured varying levels of anthropogenic disturbance, represented as the number of human structures within a specified distance from used and available locations operating at three different spatial scales (Table A models 4, 5, 6) and predicted there would be an inverse relationship between areas selected by sandhill cranes and increased density of human structures. Additionally, density of human structures at different scales might introduce disturbances that modulate diurnal habitat selection of different land-use types (Table A models 7, 8, 9). Finally, an additive relationship between land use practices, land ownership, and level of human disturbances at different spatial scales might best describe winter habitat selection by sandhill cranes during the diurnal period (Table A models 10, 11, 12). We predicted sandhill cranes would select areas with lower density of human structures, and land-use types preferred for foraging and loafing (e.g., corn fields) on public lands. See Table B for model selection results.

**Table A**. **Candidate models used to identify habitat-related features that are the most important for diurnal habitat selection by sandhill cranes during winter in the Middle Rio Grande Valley of central New Mexico.**

| Model No. | Covariates |
| --- | --- |
| 1 | land use^a^ |
| 2 | land ownership^b^ |
| 3 | land use + land ownership |
| 4 | structure density_100m^c^ |
| 5 | structure density_500m^d^ |
| 6 | structure density_1000m^e^ |
| 7 | land use + structure density_100m |
| 8 | land use + structure density_500m |
| 9 | land use + structure density_1000m |
| 10 | land use + land ownership + structure density_100m |
| 11 | land use + land ownership + structure density_500m |
| 12 | land use + land ownership + structure density_1000m |

^a^ land use = alfalfa fields, corn fields, fallow fields, small grain fields, wetlands, and an

other category. Alfalfa fields reference category.

^b^ land ownership = public lands or private lands. Private lands reference category.

^c^ structure density_100m = density of human structures within a 100 m radius of used and available locations

^d^ structure density_500m = density of human structures within a 500 m radius of used and available locations

^e^ structure density_1000m = density of human structures within a 1000 m radius of used and available locations

**Table B**. **Model selection results comparing models hypothesized to influence diurnal habitat selection by sandhill cranes on their primary wintering area, the Middle Rio Grande Valley of central New Mexico.**

| **Model No.** | **K** | **QIC** | **ΔQIC** | **w_i_** |
| --- | --- | --- | --- | --- |
| 11 | 7 | 77026.6 | 0.0 | 0.99 |
| 10 | 7 | 77036.2 | 9.6 | 0.0081 |
| 12 | 7 | 77236.8 | 210.1 | 0 |
| 9 | 6 | 77347.8 | 321.2 | 0 |
| 2 | 1 | 90196.8 | 13170.2 | 0 |
| 7 | 6 | 95805.5 | 18778.9 | 0 |
| 8 | 6 | 96532.7 | 19506.1 | 0 |
| 6 | 6 | 96814.1 | 19787.5 | 0 |
| 1 | 5 | 97747.6 | 20721.0 | 0 |
| 4 | 1 | 102003.6 | 24977.0 | 0 |
| 5 | 1 | 103136.1 | 26109.5 | 0 |
| 3 | 1 | 103278.5 | 26251.9 | 0 |

### Roosting period

As with diurnal habitat selection, we developed a candidate set of models that we hypothesized would likely influence selection of roosting habitat by sandhill cranes (Table C). When formulating these models, we did not include models with proportion of riparian vegetation for several reasons. First, proportion of riparian vegetation was almost perfectly collinear with proportion of water. There is a strong negative correlation between these variables because they are mutually exclusive. There is likely little to no water present on vegetated islands or peninsulas within the channel. Second, the physical characteristics of areas classified as riparian vegetation primarily contain built-up sedimentation that support woody vegetation and are not areas where sandhill cranes roost. The dynamic conditions of riverine systems make the physical characteristics contributing to river morphology highly interactive. Thus, we only consider models with interactive effects between geomorphic covariates (proportion of water and sandbars, and channel width) along with their main effects included in models. Although we did explore models with only additive effects of geomorphic covariates, they did not perform nearly as well as models with interactive effects so we did not include them in the final candidate model set.

To begin developing models to include in the final model set, we first ran a set of models to determine which scale of mean bank vegetation height (100 m, 500 m, 1000 m) best described habitat selection by sandhill cranes. We then only included this scale in the candidate model set for roost site selection. We took a similar approach to identify disturbance features (distance to bridge, distance to nearest human structure, and density of human structures within a 1000 m radius of used and available locations) that were most influential to roost site selection by sandhill cranes, then only included these covariates in the candidate model set. To determine the most important geomorphic characteristics influencing roosting habitat selection, we consider four subclasses of models with each subclass distinguished by interactive effects between 1) channel width and proportion water; 2) channel width and proportion of sandbars; 3) proportion of water and proportion of sandbars; and 4) channel width and proportion of water and proportion of sandbars. Within each of these subclasses, we fitted models including only these interactive effects (Table C models 1, 8, 15, 22) to identify the most important geomorphic characteristics related to roost site selection by sandhill cranes. We hypothesized the interaction between channel width, proportion of water, and proportion of sandbars would influence where sandhill cranes roost because the channel width will dictate water depth and availability of sandbars. We also included models with the additional effect of mean bank vegetation height (Table C models 2, 9, 16, 23), and distance to nearest bridge (Table C models 3, 10, 17, 24). We additionally considered several models with proximity to diurnal habitat that had a high relative probability of use (Table C models 4, 11, 18, 25). The importance of proximity of roosting and foraging areas used by sandhill cranes has been documented in other studies [1, 2]. To characterize this relationship we identified all areas that had ≥ 70% relative probability of use predicted from the most supported model for diurnal habitat selection. We classified these as suitable areas that can support sandhill cranes during the diurnal period and measured distances between these areas and used and available locations during the roosting period. We used 70% relative probability of use because it represented a conservative threshold for habitat that would likely be selected by sandhill cranes. We further considered several models that included the interactive effect of channel width and mean bank vegetation height, and hypothesized that a narrower channel would likely contain taller vertical structure of bank vegetation because of increased sediment loads which supports woody vegetation [3], and that sandhill cranes would likely avoid these areas (Table C, models 5, 12, 19, 26). Expanding on these models, we also included the additive effects of distance to bridge (Table C, models 6, 13, 20, 27), and the additive effects of both distance to bridge and diurnal habitat (Table C, models 7, 14, 21, 28). We hypothesized, in addition to important geomorphic characteristics of the Rio Grande, that sandhill cranes would roost in reaches of the Rio Grande channel farther away from anthropogenic disturbance associated with bridges (vehicle traffic and noise pollution), and proximal to diurnal habitat with a high relative probability of use. See Table D for model selection results.

**Table C**. **Candidate models used to identify habitat-related features that are most important for selection of roosting habitat in the Rio Grande by sandhill cranes during winter in the Middle Rio Grande Valley of central New Mexico.**

| **Model No.** | **Covariates** |
| --- | --- |
| 1 | channel width × water |
| 2 | channel width × water + bank veg height |
| 3 | channel width × water + distance_bridge_ |
| 4 | channel width × water + distance_habitat_ |
| 5 | channel width × water + channel width × bank veg height |
| 6 | channel width × water + channel width × bank veg height + distance_bridge_ |
| 7 | channel width × water + channel width × bank veg height + distance_bridge_ + distance_habitat_ |
| 8 | channel width × sandbar |
| 9 | channel width × sandbar + bank veg height |
| 10 | channel width × sandbar + distance_bridge_ |
| 11 | channel width × sandbar + distance_habitat_ |
| 12 | channel width × sandbar + channel width × bank veg height |
| 13 | channel width × sandbar + channel width × bank veg height + distance_bridge_ |
| 14 | channel width × sandbar + channel width × bank veg height + distance_bridge_ + distance_habitat_ |
| 15 | water × sandbar |
| 16 | water × sandbar + bank veg height |
| 17 | water × sandbar + distance_bridge_ |
| 18 | water × sandbar + distance_habitat_ |
| 19 | water × sandbar + channel width × bank veg height |
| 20 | water × sandbar + channel width × bank veg height + distance_bridge_ |
| 21 | water × sandbar + channel width × bank veg height + distance_bridge_ + distance_habitat_ |
| 22 | channel width × water × sandbar |
| 23 | channel width × water × sandbar + bank veg height |
| 24 | channel width × water × sandbar + distance_bridge_ |
| 25 | channel width × water × sandbar + distance_habitat_ |
| 26 | channel width × water × sandbar + channel width × bank veg height |
| 27 | channel width × water × sandbar + channel width × bank veg height + distance_bridge_ |
| 28 | channel width × water × sandbar + channel width × bank veg height + distance_bridge_ + distance_habitat_ |

channel width = width of Rio Grande channel

water = proportion of water within 30 m pixel of channel

sandbar = proportion of sandbars within 30 m pixel of channel

bank veg height = mean height of channel bank vegetation within a 500 m distance surrounding used/available locations

distance_habitat_ = proximity to nearest diurnal habitat with a ≥ 70% relative probability of use predicted from most supported model for diurnal habitat selection

distance_bridge_ = proximity to nearest bridge

**Table D**. **Model selection results comparing models hypothesized to influence roosting habitat selection by sandhill cranes in the Rio Grande on their primary wintering area, the Middle Rio Grande Valley of central New Mexico.**

| **Model No.** | **K** | **QIC** | **ΔQIC** | **w_i_** |
| --- | --- | --- | --- | --- |
| 27 | 10 | 14146.6 | 0.0 | 0.8183 |
| 28 | 11 | 14149.6 | 3.0 | 0.1815 |
| 26 | 9 | 14163.5 | 16.9 | 0.0002 |
| 23 | 8 | 14171.4 | 24.8 | 0 |
| 7 | 7 | 14177.3 | 30.7 | 0 |
| 6 | 6 | 14178.5 | 31.8 | 0 |
| 5 | 5 | 14189.1 | 42.5 | 0 |
| 2 | 4 | 14201.8 | 55.2 | 0 |
| 25 | 8 | 14258.9 | 112.3 | 0 |
| 22 | 7 | 14277.4 | 130.8 | 0 |
| 24 | 8 | 14279.4 | 132.8 | 0 |
| 4 | 4 | 14313.8 | 167.1 | 0 |
| 3 | 4 | 14325.1 | 178.5 | 0 |
| 1 | 3 | 14326.1 | 179.5 | 0 |
| 20 | 6 | 14341.1 | 194.5 | 0 |
| 21 | 7 | 14349.7 | 203.0 | 0 |
| 19 | 5 | 14393.3 | 246.6 | 0 |
| 17 | 4 | 14410.2 | 263.5 | 0 |
| 16 | 4 | 14410.5 | 263.8 | 0 |
| 15 | 3 | 14454.3 | 307.7 | 0 |
| 18 | 4 | 14459.9 | 313.3 | 0 |
| 13 | 6 | 16615.3 | 2468.7 | 0 |
| 14 | 7 | 16620.9 | 2474.2 | 0 |
| 12 | 5 | 16646.3 | 2499.7 | 0 |
| 9 | 4 | 16661.6 | 2515.0 | 0 |
| 10 | 4 | 16830.0 | 2683.4 | 0 |
| 8 | 3 | 16863.4 | 2716.7 | 0 |
| 11 | 4 | 16867.1 | 2720.4 | 0 |

# References

1. Krapu GL, Brandt DA, Kinzel PJ, Pearse AT. Spring migration ecology of the mid‐continent sandhill crane population with an emphasis on use of the Central Platte River Valley, Nebraska. Wildlife Monogr. 2014; 189:1-41.
2. Kinzel PJ, Nelson JM, Heckman AK. Response of sandhill crane (*Grus canadensis*) riverine roosting habitat to changes in stage and sandbar morphology. River Res Appl. 2009;25(2): 135-152.
3. Pearse AT, Krapu GL, Brandt DA. Sandhill crane roost selection, human disturbance, and forage resources. J Wildl Manage. 2017;81(3): 477-486.
